# An end-to-end computational framework for “Record-seq” transcriptional recording data

**DOI:** 10.1093/bioinformatics/btag479

**Published:** 2026-08-21

**Authors:** Florian Hugi, Tanmay Tanna, Randall J Platt

**Affiliations:** Department of Biosystems Science and Engineering, ETH Zurich, Basel, 4056, Switzerland; Department of Computer Science, ETH Zurich, Zurich, 8092, Switzerland; Department of Biosystems Science and Engineering, ETH Zurich, Basel, 4056, Switzerland; Department of Biosystems Science and Engineering, ETH Zurich, Basel, 4056, Switzerland; Department of Chemistry, University of Basel, Basel, 4056, Switzerland; Basel Research Center for Child Health (BRCCH), Basel, 4051, Switzerland; NCCR Molecular Systems Engineering, Basel, 4058, Switzerland

## Abstract

**Motivation:**

Record-seq captures cumulative transcriptional activity over time in engineered *Escherichia coli* by integrating cellular RNA-derived spacer sequences into clustered regularly interspaced short palindromic repeats (CRISPR) arrays, which are read out by sequencing. Unlike the approximately uniform transcript sampling of RNA-seq, Record-seq records biological signal as spacers sampled by the CRISPR spacer acquisition machinery. Consequently, standard RNA-seq analysis strategies are not directly applicable, limiting sensitivity and interpretability. Our previous pipeline addressed these challenges only partially, retained inherited RNA-seq assumptions, and had limited algorithmic efficiency.

**Results:**

Here, we present an end-to-end computational framework for Record-seq data. To address the primary computational bottleneck of spacer sequence extraction, we implemented a wavefront alignment approach for efficient quasi-local pattern matching, achieving an approximately 30-fold speedup. We introduce transcription unit-based feature counting as an alternative to gene-body quantification to better represent prokaryotic transcription and increase statistical power by capturing signal from untranslated regions, which are spacer acquisition hotspots. For downstream analyses, we incorporate multiple normalization strategies and a nonparametric differential expression testing framework designed for sparse datasets. Further, we analyze spacer acquisition patterns and train sequence-based neural models that predict acquisition propensity from genomic sequence and annotations, providing a framework for assessing whether acquisition rules generalize as Record-seq is extended to new microbial hosts.

**Availability and implementation:**

The primary analysis workflow, the recoRdseq package, acquisition modeling repository, and relevant data are all linked at https://github.com/plattlab/Record-seq-Framework. Acquisition models and training data are on Zenodo at https://doi.org/10.5281/zenodo.18891434.

## 1 Introduction

Clustered regularly interspaced short palindromic repeats (CRISPR)–Cas adaptation, in which CRISPR-associated proteins integrate short nucleic acid fragments as spacers into CRISPR arrays, enables bacteria and archaea to create a heritable molecular record of prior exposure to invasive genetic elements (Amitai and Sorek 2016). Record-seq leverages this process for cumulative longitudinal transcriptional recording in bacterial populations. In engineered *Escherichia coli* (*E. coli*), a heterologously expressed RT-Cas1–Cas2 complex from *Fusicatenibacter saccharivorans* reverse transcribes cellular RNA fragments and integrates them as spacers into CRISPR arrays in a site-specific manner (Schmidt *et al.* 2018). We previously showed that these engineered *E. coli* sentinel cells can capture complex transcriptional signatures associated with dietary perturbations and inflammatory states in the mammalian gut (Schmidt *et al.* 2022), establishing the potential of Record-seq for noninvasive clinical diagnostics.

From a computational perspective, Record-seq data differ fundamentally from bulk RNA-seq. First, the biological signal is encoded in short spacer sequences embedded within CRISPR arrays and requires explicit spacer identification and extraction prior to downstream analysis. Second, spacer acquisition reflects the biochemical and sequence-context preferences of CRISPR adaptation rather than approximately uniform transcript sampling. Specifically, acquired spacers are strongly enriched in untranslated regions (UTRs), including transcript boundaries and adjacent non-coding segments, rather than being distributed uniformly across annotated coding sequences (Schmidt *et al.* 2018). As a result, standard gene-body counting strategies discard a substantial fraction of informative spacers. Third, Record-seq samples are not input-normalized, hence library sizes and count distributions can differ substantially between experimental conditions, often challenging assumptions of standard normalization and differential testing frameworks.

**Figure 1:**
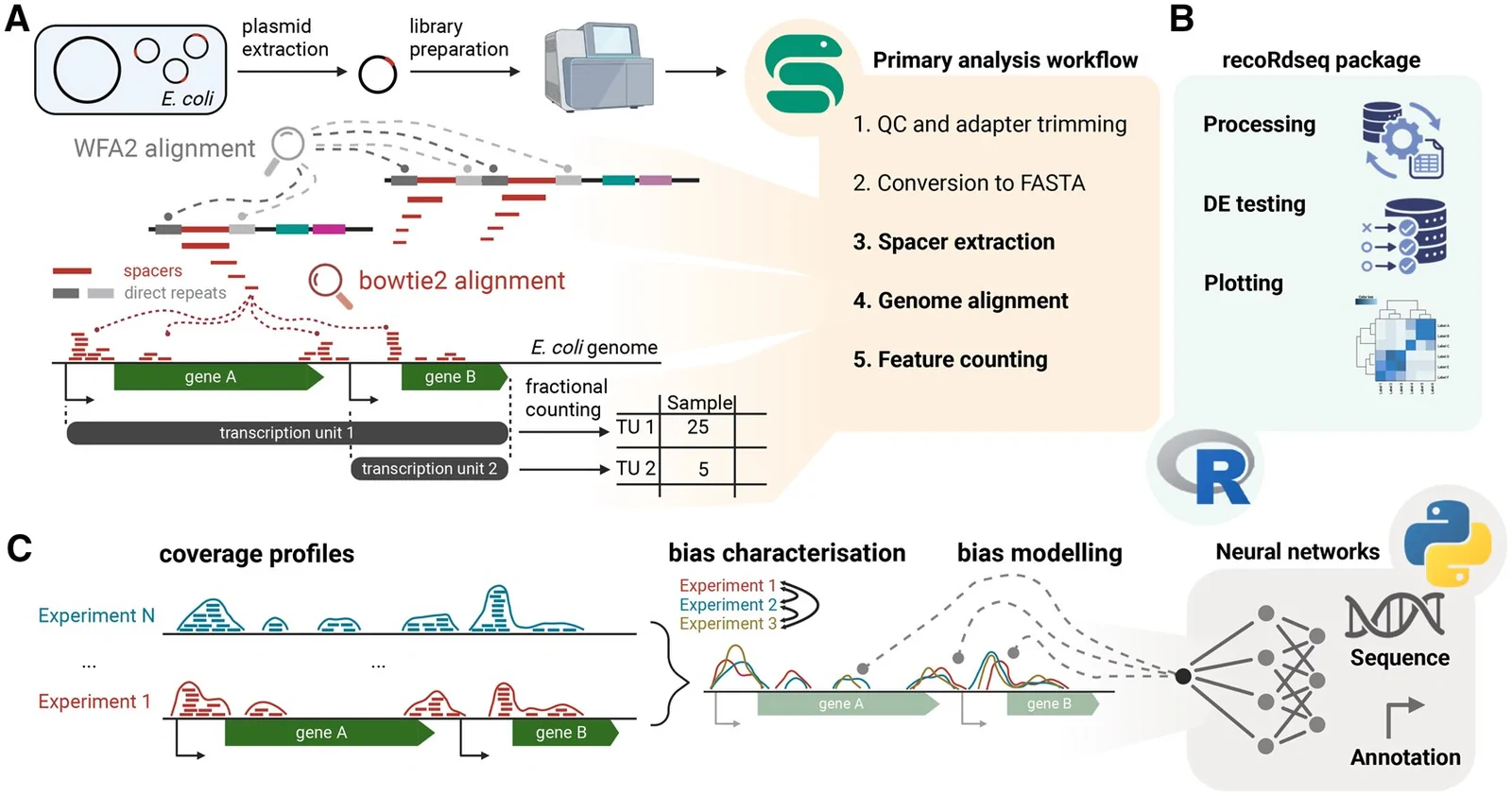
End-to-end Record-seq analysis framework. (A) Primary analysis workflow comprising read quality control, adapter trimming, conversion to FASTA, WFA2-based spacer extraction, alignment of spacers to genome references, and transcription unit (TU)-based feature counting. (B) Secondary analysis implemented in the recoRdseq R package, supporting data processing and normalization, differential expression (DE) testing, and visualization of gene- and TU-level results. (C) Characterization and modeling of spacer acquisition biases. Spacers from multiple Record-seq experiments were aligned to the *Escherichia coli* genome to derive normalized spacer coverage profiles, which were used to train sequence-based neural models that predict acquisition profiles from genomic sequence and annotations.

Building on our previously described Record-seq analysis pipeline (Tanna *et al.* 2020), we present an end-to-end computational framework for Record-seq analysis. The primary processing pipeline introduces two core advances (Fig. 1A). First, spacer extraction uses a quasi-local wavefront alignment module (Marco-Sola *et al.* 2021) that substantially improves runtime and scalability. Second, we implement transcription unit (TU)–based feature counting for *E. coli* K-12 MG1655. TUs denote contiguous strand-specific RNA units, encompassing genes together with intervening and associated untranslated transcribed sequences (Mao *et al.* 2015). This representation better reflects bacterial transcriptional organization and increases signal recovery and downstream sensitivity. Secondary analysis tools are provided in the recoRdseq package supporting multiple normalization strategies, quality control, differential expression analysis including a nonparametric approach for sparse datasets, and integrated visualization modules (Fig. 1B).

Further, we used the resulting high-resolution spacer profiles to test whether spacer acquisition patterns extend beyond previously described gene-level biases to include localized, position-specific effects within transcribed sequences (Fig. 1C). We identified pronounced acquisition hotspots that are conserved across independent Record-seq experiments, indicating that spacer sampling is shaped by intrinsic sequence and transcript-context features. We then trained sequence-based neural models to characterize and predict the genomic landscape of spacer acquisition in *E. coli*. These models predict acquisition patterns from primary sequence and genomic annotations, and provide a quantitative framework to assess whether spacer acquisition follows conserved rules as Record-seq is extended to new microbial hosts, even in regimes where recording efficiency and dataset size are initially limited.

## 2 Methods

### 2.1 Primary analysis workflow

#### 2.1.1 Workflow implementation and overview

The primary analysis workflow is implemented in Snakemake and executes reference index construction, read preprocessing, repeat-flanked spacer sequence extraction, spacer alignment to genome and plasmid references, and quantification of spacer acquisition signal. The workflow supports quantification at multiple resolutions: genomic features (genes or TUs) and individual spacer sequences, where counts are indexed by spacer identity rather than genomic coordinates. Depending on the quantification mode, the workflow applies sequence- or UMI-based spacer deduplication to remove technical amplification arising from plasmid replication and PCR while preserving biological signal. The workflow records structured status metadata for each step and compiles an aggregated diagnostic summary for common failure modes and data-quality issues, enabling rapid troubleshooting. To ensure reproducibility across computing environments, we define per-rule conda environments.

#### 2.1.2 PatternBoundExtractor: wavefront-based spacer extraction

We implemented a wavefront alignment–based module, PatternBoundExtractor, to improve the scalability of spacer extraction, the primary runtime bottleneck of Record-seq analysis. The extractor is built on the WFA2 algorithm (Marco-Sola *et al.* 2021) via the pywfa interface (Cleal 2024), which performs gap-affine alignment with time complexity O(ns), where *n* is sequence length and *s* is the optimal alignment score. This formulation yields substantial speedups for reads dominated by near-perfect matches compared to the previously used fuzzysearch-based approach (Tanna *et al.* 2020), whose runtime depends on a fixed maximum edit-distance threshold (Einat 2025). We adapted wavefront alignment to a quasi-local setting by using free end gaps and reusing aligners preconstructed for length-binned search intervals in 10-nucleotide increments. This ensures that only mismatches within the repeat contribute to the alignment score, independent of surrounding sequence, allowing consistent score thresholds across reads while keeping *s* small. Multiple spacer acquisition events are supported by iteratively splitting reads at detected repeats and applying the same extraction procedure to each segment. The overall control flow is summarized in Algorithms 1–3 in the Supplementary Methods, Section 1.1.1, available as supplementary data at *Bioinformatics* online.

#### 2.1.3 Transcription unit annotation and feature counting

Record-seq spacer acquisition is enriched in regions flanking coding sequences, including untranslated and other transcribed non-coding segments that gene-centric annotations do not capture. To align quantification with both bacterial transcript architecture and the spacer acquisition process, we implemented TU-based feature counting. We define TUs as contiguous, strand-specific transcribed regions representing RNA units (rather than isolated gene bodies), including genes and intervening transcribed segments (Mao *et al.* 2015). Since no comprehensive TU-level reference is available for *E. coli* K-12 MG1655, we constructed a custom GFF3-formatted TU annotation by integrating EcoCyc TU, gene, promoter, and terminator annotations (Karp *et al.* 2025). Where available, we anchored TU boundaries to experimentally validated transcription start sites; otherwise, we inferred TU structure from strand-consistent operon organization. We provide full annotation-construction details in the Supplementary Methods, Section 1.1.2, available as supplementary data at *Bioinformatics* online. We perform feature counting with featureCounts using fractional assignment (Liao *et al.* 2014), which ensures proportional assignment of spacers mapping to shared genomic intervals since untranslated regions, alternative promoter-defined TU variants, and nested operon structures can partially overlap.

### 2.2 recoRdseq R package for downstream analysis

The recoRdseq package provides a framework for secondary analysis of Record-seq data. It imports TU- or gene-level counts with sample metadata and supports replicate-aware preprocessing (including conservative feature filtering and within-group outlier detection), normalization and transformation, differential expression analysis, and integrated visualization (Supplementary Methods, Section 1.2, available as supplementary data at *Bioinformatics* online). To assess whether normalization adequately addresses global shifts between conditions, recoRdseq computes distributional similarity on group-level feature medians using Kolmogorov–Smirnov (KS) and Wasserstein distance metrics (Supplementary Methods, Section 1.2.3, available as supplementary data at *Bioinformatics* online). For differential expression analysis, recoRdseq integrates established frameworks run on raw count matrices (DESeq2 (Love *et al.* 2014) and edgeR (Robinson *et al.* 2010)) and also implements a sparse-data-aware Wilcoxon workflow for two-group comparisons. The Wilcoxon workflow operates on raw counts, accounts for condition-specific feature detection, normalizes retained features, and performs Wilcoxon rank-sum testing on within-sample percentile-rank transformed counts. *P*-values are calibrated via label permutations and adjusted with Benjamini-Hochberg, providing a conservative complementary inference mode for sparse data with library-size asymmetry, where parametric model assumptions may be fragile (Supplementary Methods, Section 1.2.4, available as supplementary data at *Bioinformatics* online). We evaluated the different normalization strategies using published Record-seq data, and benchmarked the Wilcoxon workflow using both downsampled published data and synthetic simulations with sparse, distributionally mismatched groups (Supplementary Methods, Section 1.3, available as supplementary data at *Bioinformatics* online).

### 2.3 Identification and characterization of local spacer acquisition biases

To characterize spacer acquisition beyond the previously described general enrichment in regions flanking gene bodies (Schmidt *et al.* 2018), we analyzed genome-wide spacer coverage at base-pair resolution across independent published Record-seq datasets (Schmidt *et al.* 2018, 2022).

#### 2.3.1 Construction of normalized spacer coverage profiles

To decouple local acquisition propensity from global differences in library size and transcript abundance, we generated normalized spacer coverage profiles. We aggregated spacer counts at each genomic position, normalized counts to a fixed library size, and locally rescaled coverage by an estimate of TU expression, enabling comparison of local acquisition patterns across TUs and experiments (Supplementary Methods, Section 1.4.1, available as supplementary data at *Bioinformatics* online).

#### 2.3.2 Quantifying similarity of normalized acquisition profiles

We assessed reproducibility of patterns by comparing normalized profiles for the same TUs across datasets. Positional concordance of acquisition hotspots was quantified using an F1 score based on genomic overlap of identified peaks in the normalized coverage profiles, while overall similarity was measured using the Spearman correlation of log-transformed profiles (Supplementary Methods, Section 1.4.2, available as supplementary data at *Bioinformatics* online).

### 2.4 Sequence-based modeling of spacer acquisition biases

We modeled local spacer acquisition biases with sequence-based neural networks at the TU level. For each TU, the model predicts a base-resolution spacer acquisition profile representing position-specific *acquirability*. We used normalized spacer coverage profiles as targets and combined profiles across experiments by library-size-weighted averaging (Supplementary Methods, Section 1.5.1, available as supplementary data at *Bioinformatics* online).

#### 2.4.1 Problem formulation, inputs, and targets

Models take as input the TU sequence together with annotation channels encoding gene bodies, promoter and terminator locations, and gene orientation. We model spacer acquisition as a Poisson process with position-specific rates (local *acquirability*) predicted by the network, and optimize model parameters by minimizing the corresponding Poisson negative log-likelihood (Kelley *et al.* 2018). We provide the formal problem formulation in Supplementary Methods, Section 1.5.2, available as supplementary data at *Bioinformatics* online.

#### 2.4.2 Training and evaluation protocol

We split data into training, validation, and test sets (70%/15%/15%) using a genome-wide TU-level split that groups TUs into connected components to prevent shared genomic sequence across splits (Supplementary Methods, Section 1.5.3, available as supplementary data at *Bioinformatics* online). We excluded highly repetitive loci, including ribosomal RNA operons and CRISPR arrays. Residual paralog families in *E. coli* are sufficiently diverged and occur in distinct regulatory contexts, making paralogy an unlikely source of train-test leakage.

We performed model selection using the training and validation sets, scoring models using loss, Spearman correlation and peak-level F1 score between predicted and observed acquisition profiles. For final evaluation, we retrained the selected model on the combined training and validation data and evaluated it on the held-out test set. Performance is reported as mean±SD across five random seeds.

#### 2.4.3 Model architecture

The selected model uses parallel sequence and annotation streams, each composed of convolutional layers followed by bidirectional long short-term memory (LSTM) layers. We concatenate the stream outputs, process them with a shared bidirectional LSTM, and apply a 1×1 convolution with rectified linear unit (ReLU) activation to produce position-specific acquisition rates. To accommodate TUs of different lengths within the same data batch, padding is added with padding-aware masking.

## 3 Results

### 3.1 Primary analysis workflow

#### 3.1.1 Runtime improvements from WFA2-based spacer extraction

**Figure 2:**
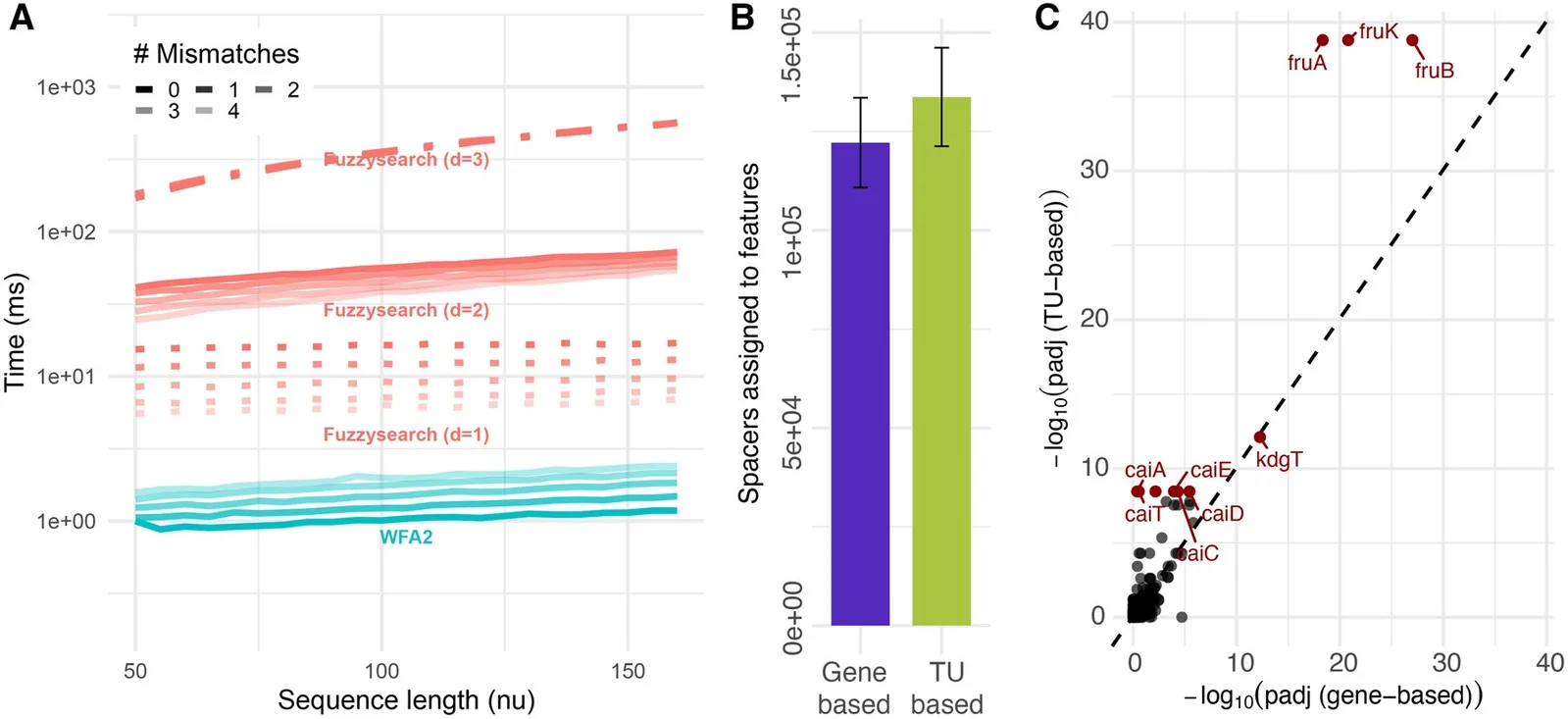
WFA2-based spacer extraction and TU-based counting improve runtime and signal detection. (A) Runtime for 1,000 spacer-alignment operations as a function of read length, comparing the original fuzzysearch-based method (edit-distance thresholds 1–3) with the WFA2-based PatternBoundExtractor for synthetic reads with 0–4 mismatches relative to the repeat pattern. Average results from 5 independent runs are shown. (B) Mean number of spacers per sample assigned to genomic features after deterministic subsampling to $10^{6}$ reads per sample, comparing gene- and TU-based counting (bars, mean; error bars, SD). (C) Comparison of statistical significance from TU- versus gene-based counting, shown as $-{\;\text{log}\;}_{10}(\text{padj})$ for all tested features. Points denote gene–TU associations (multiple genes may map to one TU); the diagonal indicates equal significance.

To benchmark spacer extraction, we measured direct-repeat identification runtimes on synthetic reads (50–160 nt; 0–4 mismatches; Fig. 2A). In the original fuzzysearch-based implementation, runtime was primarily determined by the maximum edit-distance threshold, so permissive settings remained computationally expensive even when reads had few errors. The WFA2-based PatternBoundExtractor, in contrast, scales with read length and observed alignment score. Compared to the original pipeline setting (fuzzysearch with maximum edit distance = 2), it achieved 30–60× speedups for reads without mismatches and 27–34× speedups for reads with two mismatches. Our quasi-local wavefront formulation, in which free end gaps and reuse of aligners preconstructed for length-binned intervals restrict the alignment score to mismatches within the repeat, drove these gains, and omitting this binning strategy substantially increased runtime (Fig. 1, available as supplementary data at *Bioinformatics* online).

We re-analyzed the dietary intervention Record-seq dataset from Schmidt *et al.* (2022) (240 samples; 400,212,561 reads) on a standard laptop (MacBook Pro, November 2024; Apple M4; 16 GB memory). The full end-to-end analysis completed in ∼2 hours, demonstrating practical local processing of full-scale Record-seq datasets.

#### 3.1.2 TU-based feature counting increases signal recovery and statistical power

To evaluate how TU-based feature counting affects spacer utilization and downstream inference, we reanalyzed the dietary intervention dataset (Schmidt *et al.* 2022) and focused on the last time point (Day 20), where transcriptional differences between experimental groups are weakest. At matched sequencing depth, TU-based counting consistently assigned more spacers per sample than gene-based counting (Fig. 2B), indicating improved signal recovery.

We next compared downstream differential expression analysis between dietary conditions using DESeq2 (Wald test with FDR correction). TU-based testing generally produced lower adjusted *P*-values than gene-based methods (Fig. 2C) by increasing effective counts and reducing the multiple-testing burden. This benefit was most pronounced in multi-gene TUs like *fruABK*, where signal aggregation outperformed individual gene testing (*fruA*, *fruB*, *fruK*); conversely, single-gene TUs like *kdgT* showed comparable significance. However, TU-level analysis can reduce sensitivity in nested or overlapping units, where fractional assignment dilutes signal when differential expression is restricted to a subset of the TU. For example, *recA* significance dropped (*P*_adj_ = 0.01 to 0.03) when analyzed within the larger *recAX* TU, which included the non-differentially abundant *recX*. Overall, these results show that TU-based feature definitions substantially improve effective spacer utilization and typically increase statistical power in Record-seq analyses.

### 3.2 Normalization strategies and sparse-data differential testing in recoRdseq

**Figure 3:**
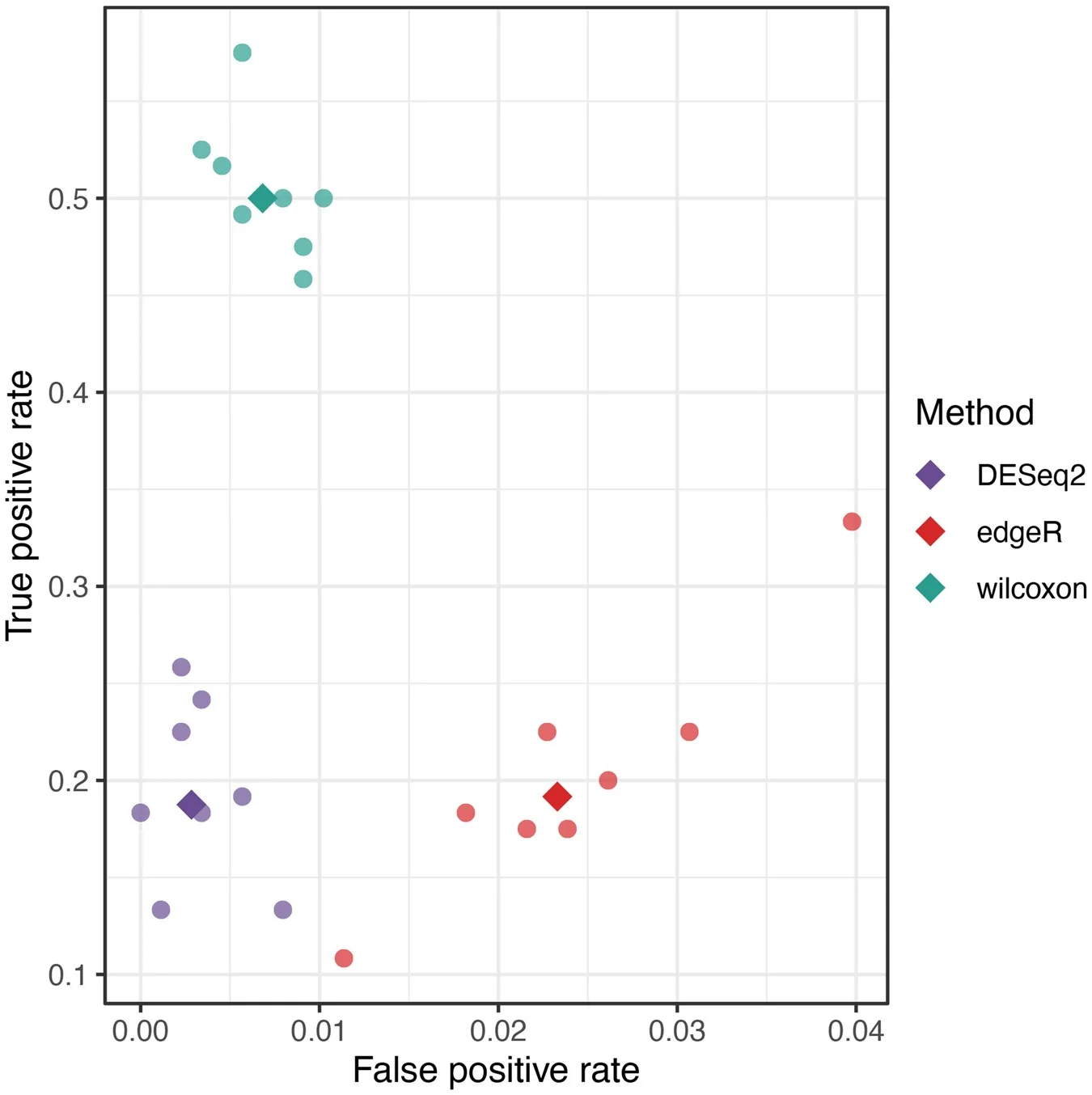
Simulation benchmark for sparse, distributionally mismatched groups. TPR is plotted against FPR comparing DESeq2 (Wald), edgeR (quasi-likelihood), and the recoRdseq Wilcoxon workflow across 10 independent simulations. Benchmarking was performed on 2-group datasets featuring dropouts, asymmetric library sizes, and outliers (Supplementary Methods, Section 1.3.3, available as supplementary data at *Bioinformatics* online).

Using heuristic distribution-similarity diagnostics on published and simulated count matrices, rlog from DESeq2 (Love *et al.* 2014) produced the strongest alignment of group-level feature-median distributions in this dataset and is therefore used as the default transformation for visualization and QC in recoRdseq (Fig. 2A, available as supplementary data at *Bioinformatics* online; Supplementary Results, Section 2.2.1, available as supplementary data at *Bioinformatics* online). In a deliberately challenging sparse dataset designed to induce distributional mismatch (dropouts, library-size asymmetry, and outliers), the Wilcoxon workflow achieved the highest TPR at a FPR comparable to DESeq2, whereas edgeR showed elevated FPR (Fig. 3; Supplementary Results, Section 2.2.4, available as supplementary data at *Bioinformatics* online). Across 10 independent downsampling trials of real data with induced distributional mismatch, the Wilcoxon-based workflow maintained the lowest FPR (Fig. 2C, available as supplementary data at *Bioinformatics* online), supporting its use as a conservative complementary workflow for sparse applications where minimizing false positives is critical, such as clinical datasets.

### 3.3 Spacer acquisition exhibits conserved local hotspots across experiments

**Figure 4:**
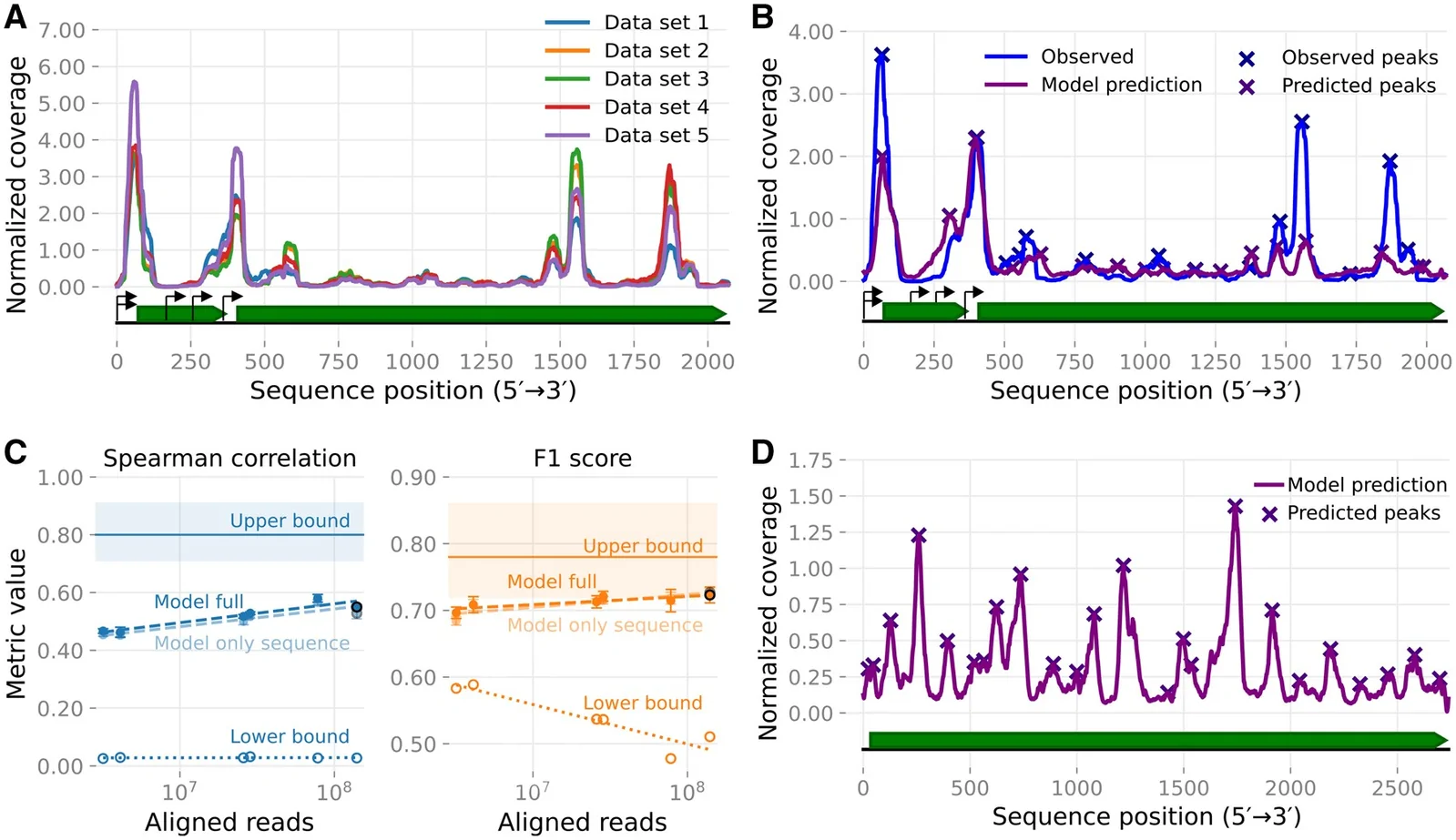
Conserved local acquisition hotspots and sequence-based modeling. (A) Normalized spacer acquisition profiles across five independent Record-seq experiments for the *groSL* TU. Rectangles and arrows indicate gene bodies and promoters, respectively. (B) Cross-experiment average normalized coverage profile and model prediction with called hotspots indicated by crosses. Shown TU was held out from training. (C) Test-set performance of the final model versus dataset size (Spearman correlation of log-normalized profiles; peak-level F1); the upper bound is derived from cross-experiment agreement (line: mean, shaded area: range), dots indicate mean ± SD (n=5 training runs) for the test set. The outlined point indicates the model trained on the combined datasets. (D) Example model-predicted acquisition profile for a *Bacteroides thetaiotaomicron* TU, illustrating cross-species application of acquisition rules learned in *Escherichia coli*.

To assess spacer acquisition preferences beyond the previously described 5' and 3' UTR enrichment observed in aggregated gene-level profiles, we compared normalized coverage profiles for the same TUs across independent Record-seq experiments, including both *in vitro* cultures and *in vivo* mouse-gut sentinel recordings. Local acquisition patterns were highly conserved across experiments (Fig. 4A), with mean Spearman correlation 0.80 (range 0.71–0.91) and mean peak-level F1 score 0.78 (range 0.72–0.86). As a baseline, comparisons between unrelated TUs within the same experiment yielded substantially lower similarity (mean peak-level F1 = 0.53; mean Spearman correlation = 0.032), indicating that cross-experiment agreement far exceeds chance expectations.

### 3.4 Sequence-based models recover acquisition hotspots and profile structure

We tested whether genomic sequence and transcriptional annotations are sufficient to recover local spacer acquisition patterns by training neural networks to predict normalized spacer coverage profiles averaged across datasets. Recurrent models (convolutional layers followed by bidirectional LSTMs) substantially outperformed convolution-only baselines, indicating that local sequence motifs alone are insufficient to localize acquisition hotspots, while adding dot-product attention provided no further benefit, suggesting limited dependence on long-range interactions.

On held-out TUs, the selected model recovered localized acquisition hotspots with a peak-level F1 score of 0.7231±0.0119 across five random seeds, substantially exceeding the random-pairing baseline and approaching the empirical upper bound given by cross-experiment agreement. It also captured overall profile structure (Spearman correlation 0.5499±0.0083; Fig. 4B and C), performing well above chance but remaining below the empirical upper bound. Model interpretation and architectural ablations revealed that acquisition propensity is primarily governed by local base composition—specifically AT-richness—rather than discrete motifs, and recurrent layers are essential to integrate these short-range features for precise hotspot localization. Additional architecture ablations and sequence-feature sensitivity analyses are provided in Supplementary Results, Section 2.3.1, available as supplementary data at *Bioinformatics* online. Sequence-only training yielded nearly identical performance scores across datasets (F1: 0.7268±0.0071 and Spearman 0.5284±0.0192 for combined dataset), indicating that primary sequence dominates hotspot localization while transcriptional context provides only secondary refinement (Fig. 4C).

Finally, applying the model to the *Bacteroides thetaiotaomicron* genome (Xu *et al.* 2003), we generated exploratory base-resolution acquisition profiles as a reference for non-*E. coli* hosts (Fig. 4D). Because *E. coli* data indicate that spacer acquisition predominantly reflects mRNA-level transcriptional activity, these unvalidated *B. thetaiotaomicron* predictions provide hypotheses for testing whether learned acquisition rules generalize across species or show systematic, species-specific deviations.

## 4 Discussion

We present a computational framework for Record-seq that incorporates TU-based feature definitions, wavefront-based spacer extraction, and sparse-data-aware downstream processing to improve signal utilization for genome-wide spacer analysis. TU-level spacer coverage provides a position-resolved view of acquisition within transcripts and reveals localized hotspots that are reproducible across independent experiments, supporting a model in which spacer acquisition is shaped not only by transcript abundance and experimental context, but also by intrinsic properties of sequence and transcript organization. Consistent with this, sequence-based neural models capture these biases directly from genomic information, establishing a quantitative framework for predicting acquisition profiles and for testing whether acquisition rules generalize to additional microbial hosts.

Beyond Record-seq, PatternBoundExtractor addresses a broader and frequently occurring sequence-parsing problem: recovery of an unknown insert flanked by known sequence elements that may contain mismatches (and limited indels). This setting arises across many biological assays in which conserved handles, repeats, adapters, or barcodes flank variable sequence content. In this regime, wavefront based quasi-local alignment with free end gaps provides an efficient and robust alternative to fixed-threshold approximate matching, and should therefore be transferable to a broad class of extraction tasks beyond CRISPR spacer recovery.

The TU-based framework is likewise relevant beyond Record-seq. The *E. coli* TU reference generated here from EcoCyc annotations and explicit construction rules provides a reusable resource for transcriptomics analyses that aim to represent operon structure and untranslated transcribed regions more faithfully than gene-based references. A direct next step is to benchmark this reference on conventional *E. coli* RNA-seq datasets and test whether TU-level counting improves downstream inference, including differential expression sensitivity, effect-size stability, and biological interpretability in loci with overlapping or multi-gene transcriptional organization.

Similarly, the recoRdseq package, by combining preprocessing, heuristic normalization diagnostics, and complementary parametric and nonparametric testing, provides a practical analytical framework for sparse, distributionally mismatched data that is not inherently restricted to Record-seq.

For the modeling analyses, acquisition profiles were normalized to remove transcript-level expression effects and isolate local, position-specific acquisition biases within TUs. Because no absolute ground-truth expression measurements were available, transcript abundance had to be inferred from Record-seq data itself, necessitating a normalization that equalizes mean coverage across TUs. As a consequence, the training targets preserve relative acquisition structure within transcripts but do not encode global differences in overall transcript-level *acquirability*. The current models therefore learn the hotspots within a transcript from which spacers are preferentially acquired, but not which transcripts are sampled more efficiently in absolute terms.

A natural next step is to perform paired Record-seq and RNA-seq experiments under controlled conditions (for example, in turbidostat cultures). Independent expression measurements would enable direct estimation of transcript-level acquisition bias scores by comparing observed Record-seq signal to steady-state transcript abundance. Such bias estimates could support correction of Record-seq counts, improving quantitative comparability across TUs and enabling future models to jointly learn local acquisition structure and global transcript-level sampling efficiency, thereby strengthening inference about how faithfully Record-seq reflects absolute transcriptional activity.

Overall, this work advances Record-seq analysis by aligning computational approaches with recording biology, uncovering conserved acquisition biases, and establishing a modeling framework that supports principled extension of transcriptional recording beyond its current experimental scope, while also contributing reusable methods and references for bacterial sequencing data analysis more broadly.

## Supplementary Material

btag479_Supplementary_Data

## Data Availability

The source code for the primary analysis workflow, the recoRdseq package, and the spacer acquisition modeling framework, along with processed count matrices and instructions for retrieving raw sequencing data from the Sequence Read Archive (SRA), are available at https://github.com/plattlab/Record-seq-Framework. Trained acquisition models and training datasets have been deposited in Zenodo at https://doi.org/10.5281/zenodo.18891434.
