# Supplementary material for: An end-to-end computational framework for “Record-seq” transcriptional recording data
Source: Bioinformatics. 2026 Aug 21;42(Suppl 2):btag479. doi: 10.1093/bioinformatics/btag479 (PMC13501316; doi:10.1093/bioinformatics/btag479)
Supplement: Supplementary file 1 [file btag479_supplementary_data.pdf]

S1. Supplementary Methods

S1.1. Implementation details of the primary analysis pipeline

S1.1.1. Algorithmic details of wavefront-based spacer extraction

Algorithms 1–3 below detail the wavefront-based procedure used to identify and extract repeat-flanked spacers from sequencing reads. Reads are first filtered by length and, when configured, by the presence of the expected library barcode. For reads that may contain multiple spacer acquisitions, internal full-repeat matches are used to split the read into subreads, so that each subread contains at most one candidate spacer. Spacer extraction is then performed on each read or subread by identifying the two flanking repeat patterns and returning the intervening sequence if it satisfies the configured constraints.

P_1 and P_2 denote the known upstream and downstream repeat sequences flanking the spacer. P_{full} denotes the full repeat sequence used to identify internal repeat boundaries in reads containing multiple spacers. $K_{\max}$ is the maximum number of spacers extracted per read; $K_{\max} > 1$ enables splitting a read into subreads before applying single-spacer extraction. $L_{\min}$ and $L_{\max}$ define accepted read-length bounds. LBC denotes an optional library barcode or any other sequence that needs to be present at a specific position in a read as a prerequisite for processing; CheckLBC discards reads lacking the expected barcode. When unique molecular identifiers (UMIs) are included in the experimental design, ApplyUMICollapse can be used in addition to standard sequence-based deduplication to collapse candidate spacers with matching UMIs and reduce PCR-derived duplicates.

For each pattern P, $\mathcal{A}_P[\ell]$ is a precomputed WFA2 aligner for length bin ℓ. RoundToBin maps a sequence length to the nearest available bin, here in 10-nt increments. Exact pattern matching is attempted first. The WFA2 aligner is used only when no exact match is found for the corresponding pattern. In that case, $\mathcal{A}_P[\ell]$.align(seq) returns the best quasi-local alignment of P to seq, including its score and query coordinates. The pattern-specific thresholds τ_{full}, τ_1, and τ_2 define the minimum accepted alignment scores for P_{full}, P_1, and P_2, respectively; these thresholds were calibrated to the expected sequencing and PCR error rates of the Record-seq experimental workflow and to match the cutoffs used in the previous pipeline. LenOK(aln, P) checks whether the aligned interval length is compatible with the expected length of pattern P, thereby filtering partial or spuriously extended matches. For P_1, either the exact-match end coordinate or, if necessary, aln.end, defines the inferred spacer start. For P_2, either the exact-match start coordinate or, if necessary, aln.start, defines the inferred spacer end. A spacer is retained only if both coordinates are valid and the inferred spacer length satisfies the configured constraints.

Algorithm 1 `PatternBoundExtractor`: Main Workflow

Require: Input reads $\mathcal{R}$; Patterns $P_1, P_2, P_{\text{full}}$; Max spacers $K_{\max}$; Read length constraints $L_{\min}, L_{\max}$; Library barcode LBC
Ensure: Set of unique extracted spacers $\mathcal{S}$ with counts

Initialize WFA aligners $\mathcal{A}_P[\ell]$ for $P \in \{P_1, P_2, P_{\text{full}}\}$ categorized by length bins $\ell \in \{L_{\min}, L_{\min}+10, \ldots, L_{\max}\}$
for all $r \in \mathcal{R}$ **do**
    if $|r| \notin [L_{\min}, L_{\max}]$ **then**
        continue
    end if
    if LBC enabled **and** CheckLBC(r) is False **then**
        continue
    end if
    $Candidates \leftarrow$ List()
    if $K_{\max} > 1$ **then** ▷ **Multi-spacer extraction**
        $Subreads \leftarrow$ ExtractSubreads(r, P_{full})
        for all $sub \in Subreads$ **do**
            $s \leftarrow$ ExtractSpacer(sub, P_1, P_2)
            if $s \neq$ null **then**
                $Candidates$.append(s) ▷ Index ≡ Position
            end if
        end for
    else ▷ **Single-spacer extraction**
        $s \leftarrow$ ExtractSpacer(r, P_1, P_2)
        if $s \neq$ null **then**
            $Candidates$.append(s)
        end if
    end if
    if UMIs enabled **and** $Candidates \neq \emptyset$ **then**
        ApplyUMICollapse($Candidates$)
    end if
    $\mathcal{S} \leftarrow \mathcal{S} \cup Candidates$
end for

Algorithm 2 Subread Extraction

function ExtractSubreads(r, P_{full})
    $Matches \leftarrow$ Find all exact occurrences of P_{full} in r
    while $|Matches| < (K_{\max} - 1)$ **and** gaps exist **do**
        $g \leftarrow$ Sequence of gap $> L_{\min}$ between sorted $Matches$
        $\ell \leftarrow$ RoundToBin($|g|$)
        $aln \leftarrow \mathcal{A}_{P_{\text{full}}}[\ell]$.align($g$)
        if aln.score $> \tau_{\text{full}}$ **and** LenOK(aln, P_{full}) **then**
            $Matches \leftarrow Matches \cup aln$
        end if
    end while
    $Subreads \leftarrow$ Split r at boundaries of sorted $Matches$
    return $Subreads$
end function

Algorithm 3 Spacer Extraction

```
function ExtractSpacer(seq, P_1, P_2)
    ℓ ← RoundToBin(|seq|)
    start ← Find exact P_1;    end ← Find exact P_2
    if start is null then
        aln ← A_{P_1}[ℓ].align(seq)
        if aln.score > τ_1 and LenOK(aln, P_1) then
            start ← aln.end
        end if
    end if
    if end is null then
        aln ← A_{P_2}[ℓ].align(seq)
        if aln.score > τ_2 and LenOK(aln, P_2) then
            end ← aln.start
        end if
    end if
    if start, end valid and distance within constraints then
        return seq[start : end]
    end if
    return null
end function
```

S1.1.2. Construction of Transcription unit annotations and feature counting

TU annotations for *E. coli* K-12 MG1655 were constructed by integrating EcoCyc annotations for TUs, genes, promoters, and terminators. Promoters were represented by their annotated transcription start site, denoted p_{+1}. Each TU was represented as a strand-specific genomic interval,

$$\mathrm{TU}_i = (s_i, e_i, \sigma_i), \qquad \sigma_i \in \{+, -\}, \tag{1}$$

where s_i and e_i denote the lower and upper genomic coordinates of TU i, respectively, and σ_i denotes its strand.

When an experimentally annotated promoter was available, TU boundaries were anchored at the absolute transcription start site,

$$s_i \leftarrow p_{+1} \ \ (\sigma_i = +), \qquad e_i \leftarrow p_{+1} \ \ (\sigma_i = -), \tag{2}$$

thereby aligning TU coordinates with transcription initiation and excluding upstream regulatory regions, i.e. non-transcribed promoter-proximal sequence upstream of the transcription start site.

For TUs lacking explicit promoter annotations, candidate promoters were assigned by name-based matching of the first gene of the TU to nearby promoters on the same strand, requiring

$$|p_{+1} - b_i| < 500 \text{ bp}, \qquad \sigma(p_{+1}) = \sigma_i, \tag{3}$$

where b_i denotes the strand-proximal boundary of TU i, and $\sigma(p_{+1})$ denotes the strand of the promoter. If no suitable promoter was identified, the original EcoCyc TU boundaries were retained.

Genes not represented in any TU were incorporated either as standalone TUs or merged into existing TUs when fully spanned and strand-consistent. Genes overlapping multiple TUs on opposing strands were retained as separate units. When terminator annotations were unavailable, TU boundaries were conservatively extended by a fixed offset,

$$e_i \leftarrow e_i + 15 \text{ bp} \ \ (\sigma_i = +), \qquad s_i \leftarrow s_i - 15 \text{ bp} \ \ (\sigma_i = -), \tag{4}$$

to approximate transcript termination.

Two versions of the TU reference were generated. In the *full* annotation, alternative promoter-defined TU variants were retained as separate features. In the *collapsed* annotation, TU variants corresponding to the same operon (i.e. sharing gene composition and strand) were merged into a single representative spanning the maximal genomic extent,

$$\mathrm{TU}_{\text{collapsed}}(g) = \big(\min_{i \in \mathcal{I}(g)} s_i, \ \max_{i \in \mathcal{I}(g)} e_i, \ \sigma\big), \tag{5}$$

where $\mathcal{I}(g)$ indexes promoter-defined TU variants associated with operon g, and σ denotes their shared strand.

Feature counting was performed using `featureCounts` with fractional assignment enabled to account for overlapping untranslated regions, alternative promoter usage, and nested TU structures.

S1.2. R package for downstream analysis

S1.2.1. `recoRdseq` package overview and data model

In `recoRdseq`, the central data structure is a Bioconductor `SummarizedExperiment`. Genome-aligned spacer counts are stored as the `genomeCounts` assay, sample metadata as `colData`, and feature annotations as `rowData`. Downstream normalization and transformation append an additional assay, `normCounts` (added by `recoRdseq.transform()`), which stores $\log_2$-scale normalized/transformed values.

Input data are assembled using `recoRdseq.input()`, which accepts either explicit file paths or an input directory (auto-discovering `genomeCounts.txt` and associated metadata/summary files). The helper harmonizes sample identities across the counts matrix and user-provided metadata (and optional plasmid and per-sample summary inputs), retaining only the intersection of sample identifiers and dropping mismatches with warnings. If provided, plasmid-aligned feature counts are stored in `metadata()` and are additionally attached as a second assay (`plasmidCounts`) only when their feature identifiers match those of `genomeCounts`.

S1.2.2. Preprocessing and replicate-aware outlier detection

Because Record-seq library sizes can vary substantially across samples, `recoRdseq.preprocess()` performs depth-based quality control prior to downstream inference. Features with zero counts across all samples are removed. For each sample i, the genome-aligned library size is

$$C_i = \sum_{g=1}^{G} x_{g,i}, \tag{6}$$

where $x_{g,i}$ is the raw count for feature g in sample i and G is the number of features. Samples with $C_i < C$ are removed (`min_sample_counts`; default $C = 5000$; we recommend $C \geq 10000$ when feasible).

Outlier detection is then performed within replicate groups on $\{C_i\}$ values. Replicate groups can be specified explicitly via `group_by` (one or more `colData` columns). If `group_by` is not provided, `recoRdseq` selects suitable non-numeric metadata columns with repeated values and uses unique column combinations to define replicate groups. Within-group outlier detection is applied only for groups with at least m samples (`min_samples_per_group`; default $m = 3$).

For a replicate group r, let $\mathbf{C}_r$ denote the vector of corresponding C_i values. By default, `recoRdseq` computes a robust

z-score using the group median $\tilde{C}_r$ and median absolute deviation MAD_r (`outlier_z_method = "median"`):

$$Z_i = \frac{C_i - \tilde{C}_r}{1.4826 \cdot \mathrm{MAD}_r}. \tag{7}$$

Optionally, mean/standard-deviation standardization can be used (`outlier_z_method = "mean"`). If $\mathrm{MAD}_r = 0$ (or $\mathrm{sd}(\mathbf{C}_r) = 0$), z-scores are set to 0 for that group.

A sample is marked as a low-depth outlier if (i) $Z_i < -|z_{\max}|$ (`outlier_z_threshold`; default $z_{\max} = 3$) and (ii) it is depleted relative to the group mean $\mu_r = \mathrm{mean}(\mathbf{C}_r)$ by at least a fraction δ:

$$\frac{\mu_r - C_i}{\mu_r} \geq \delta \qquad (\texttt{outlier\_min\_deficit\_fraction};\ \delta = 0.25). \tag{8}$$

By default, only low-depth outliers are removed (`outlier_side = "low"`); high-depth outliers can additionally be removed with `outlier_side = "both"`. Removed sample identifiers are recorded in `metadata()` for provenance.

S1.2.3. Normalization, transformation, and distributional similarity diagnostics

After filtering, `recoRdseq.transform()` appends a transformed matrix as a `normCounts` assay. Implemented options include: direct log transform (`"log2"`, $\log_2(x+1)$); total-count scaling to the median library size followed by $\log_2(\cdot + 1)$ (`"totalCount"`); `DESeq2`-based size-factor normalization and transformations (`"sizeFactorNorm"`, `"vst"`, `"rlog"`; with design ∼`normalize_by` when metadata are present and `blind=FALSE`); and `edgeR`-based TMM normalization with $\log_2(\mathrm{CPM}+1)$ (`"tmm"`). `recoRdseq.transform()` defaults to `method = "rlog"`, which is often conservative in sparse settings (at the cost of runtime on large n); as in `DESeq2`, `rlog` values can be negative. The package also supports optional downsampling to the minimum observed library size via binomial thinning (`"thinCounts"`). When plasmid counts are available, genome-aligned counts can additionally be scaled by plasmid features such as RT-Cas1 or Cas2 and rescaled to the median plasmid-feature count across samples (`"rtcas1"`, `"cas2"`).

To assess between-group distributional comparability after normalization, `recoRdseq.transform()` computes two complementary distribution-similarity diagnostics for all pairwise group comparisons defined by `normalize_by` (or the first `colData` column when `normalize_by = NULL`). Let $T_{g,i}$ denote the transformed value for feature g in sample i (the `normCounts` assay). For each group a, gene-level group medians are computed as

$$m_{g,a} = \mathrm{median}_{i \in a}(T_{g,i}), \tag{9}$$

yielding a vector $\mathbf{m}_a = (m_{1,a}, \ldots, m_{G,a})$ for each group. For each pair of groups (a, b), `recoRdseq` computes:

Kolmogorov–Smirnov statistic (maximum gap).
Defining empirical CDFs $F_a(t) = \frac{1}{G}\sum_{g=1}^{G} \mathbb{I}\{m_{g,a} \leq t\}$ and $F_b(t)$ analogously, the KS statistic is

$$D_{ab} = \sup_{t \in \mathbb{R}} |F_a(t) - F_b(t)|. \tag{10}$$

First-order Wasserstein distance (L_1 earth mover's distance; average shift).
Let $m_{(1),a} \leq \cdots \leq m_{(G),a}$ and $m_{(1),b} \leq \cdots \leq m_{(G),b}$ denote the sorted group-median vectors. The implemented one-dimensional Wasserstein-1 distance with uniform weights is

$$\mathrm{EMD}_{ab} \;=\; \frac{1}{G}\sum_{j=1}^{G} \left| m_{(j),a} - m_{(j),b} \right|. \tag{11}$$

Because all `recoRdseq` normalization paths return $\log_2$-scale data, EMD_{ab} can be interpreted as an average absolute $\log_2$ shift; in particular, $\mathrm{EMD}_{ab} > 0.3$ corresponds to an average multiplicative shift greater than $2^{0.3} \approx 1.23\times$ on the original scale.

A warning is issued if any group pair satisfies $D_{ab} > 0.25$ or $\mathrm{EMD}_{ab} > 0.3$; these diagnostics serve as QC signals (not automatic method switching) to identify normalization choices that leave groups poorly aligned.

S1.2.4. Differential expression analysis

Differential expression testing is performed by `recoRdseq.DE()`, which operates on the raw `genomeCounts` assay and stores results in `rowData` and `metadata()` for downstream visualization and export. The package provides wrappers around `DESeq2` (Love et al. 2014) (Wald or LRT tests; optional $\log_2$ fold-change shrinkage; independent filtering enabled by default) and `edgeR` (Robinson et al. 2010) (quasi-likelihood workflow with `glmQLFit`/`glmQLFTest`; Benjamini–Hochberg adjustment).

Wilcoxon-based workflow for sparse, distributionally mismatched groups.
For two-group comparisons in sparse datasets where one condition has a markedly higher zero fraction (and the post-normalization distribution-similarity diagnostics indicate poor alignment), `recoRdseq` implements a Wilcoxon rank-sum workflow (`de_methods = "wilcoxon"`). Let the two groups be G_1 and G_2 with sizes n_1 and n_2 ($n = n_1 + n_2$), and let $x_{g,i}$ denote raw counts.

(i) Identify the globally sparse group and exclude low-information features on raw counts. Define the group-level zero fractions

$$z_k \;=\; \frac{1}{G\,n_k}\sum_{g=1}^{G}\sum_{i \in G_k} \mathbb{I}\{x_{g,i} = 0\}, \qquad k \in \{1,2\}. \tag{12}$$

The *globally sparse group* is the group with larger z_k (ties yield no sparse-group designation). Genes with all-zero counts in both groups are excluded:

$$\mathcal{E}_0 \;=\; \left\{ g : \sum_{i \in G_1} \mathbb{I}\{x_{g,i} = 0\} = n_1 \;\wedge\; \sum_{i \in G_2} \mathbb{I}\{x_{g,i} = 0\} = n_2 \right\}. \tag{13}$$

If a sparse group exists, an additional exclusion is applied to genes that are all-zero *only* in the sparse group. Within each sample i, genes are ranked by their raw counts across features and converted to within-sample percentile ranks

$$Q_{g,i} \;=\; \frac{\mathrm{rank}(x_{g,i}; \{x_{1,i}, \ldots, x_{G,i}\}) - 1}{G - 1} \in [0,1], \tag{14}$$

using average ranks for ties. Let $\tilde{Q}_g = \mathrm{median}_{i \in G_{\text{non-sparse}}}(Q_{g,i})$ be the median percentile rank in the non-sparse group. For genes that are all-zero in the sparse group but not all-zero in the other group, `recoRdseq` excludes genes with $\tilde{Q}_g \leq p$ where p = `wilcoxon_exclude_percentile`/100 (default $p = 0.75$). A high-rank rescue retains genes with $\tilde{Q}_g \geq 0.85$ (top 15%) unless

sparse-group detection is extremely shallow, quantified by the sparse detection fraction

$$d_{\mathrm{sparse}} = 1 - z_{\mathrm{sparse}}, \tag{15}$$

in which case rescue is disabled when $d_{\mathrm{sparse}} \leq 0.20$.

(ii) Estimate normalization factors after exclusion. Normalization is estimated using only non-excluded genes. By default, `DESeq2` size factors s_i are estimated with `type = "poscounts"` on the retained gene set (design ~ 1), and normalized counts are

$$\tilde{x}_{g,i} = \frac{x_{g,i}}{s_i}. \tag{16}$$

If size-factor estimation fails, `recoRdseq` falls back to median-scaled library-size normalization computed on non-excluded genes:

$$\tilde{x}_{g,i} = x_{g,i}\,\frac{\mathrm{median}_j(C_j^*)}{C_i^*}, \qquad C_i^* = \sum_{g\in\mathcal{S}} x_{g,i}, \tag{17}$$

where $\mathcal{S}$ is the set of genes used for normalization.

(iii) Report effect size on normalized counts. Wilcoxon effect sizes are reported as a $\log_2$ fold change based on group medians with pseudocount $c = 0.5$:

$$\log_2 \mathrm{FC}_g = \log_2(\mathrm{median}_{i\in G_2}(\tilde{x}_{g,i}) + c) - \log_2(\mathrm{median}_{i\in G_1}(\tilde{x}_{g,i}) + c). \tag{18}$$

(iv) Rank-based testing with shared-label permutation calibration. To reduce sensitivity to scale differences and sparse outliers, normalized counts are converted within each sample to scaled ranks across genes:

$$R_{g,i} = \frac{\mathrm{rank}(\tilde{x}_{g,i}; \{\tilde{x}_{1,i}, \dots, \tilde{x}_{G,i}\}) - 1}{G-1} \in [0,1], \tag{19}$$

with average ranks for ties. For each gene g, the Wilcoxon rank-sum statistic is computed by ranking $\{R_{g,i}\}_{i=1}^n$ across samples and summing the ranks in G_2:

$$W_g = \sum_{i\in G_2} \mathrm{rank}(R_{g,i}; \{R_{g,1}, \dots, R_{g,n}\}), \qquad \mu_W = \frac{n_2(n+1)}{2}. \tag{20}$$

Permutation-calibrated two-sided p-values are then computed using a *shared* set of group-label permutations reused across all genes for efficiency. Let $\{G_2^{(b)}\}_{b=1}^B$ denote either (a) exact enumeration of all labelings when $\binom{n}{n_2}$ is sufficiently small, or (b) a sampled set of B = `wilcoxon_n_permutations` random labelings (default $B = 1000$; `wilcoxon_permutation_seed` controls reproducibility). For each gene, the permutation p-value is

$$p_g = \frac{1 + \sum_{b=1}^B \mathbb{I}\left\{\left|W_g^{(b)} - \mu_W\right| \geq |W_g - \mu_W|\right\}}{B+1}, \qquad W_g^{(b)} = \sum_{i\in G_2^{(b)}} \mathrm{rank}(R_{g,i}; \{R_{g,1}, \dots, R_{g,n}\}). \tag{21}$$

P-values are adjusted across tested genes using Benjamini–Hochberg FDR. For transparency, `recoRdseq` additionally records the standard asymptotic `wilcox.test` p-value computed on $\{R_{g,i}\}$ (with `exact = FALSE`) alongside the permutation-calibrated p-value used for inference.

S1.3. `recoRdseq` data reanalysis and controlled benchmarks

S1.3.1. Reanalysis of the published Schmidt et al. Record-seq dataset

The `recoRdseq` package bundles an example dataset corresponding to day 7 of the mouse dietary intervention experiment reported in Schmidt et al. (2022), which we re-analyze here to demonstrate normalization choice and distributional comparability assessment. We used `recoRdseq.preprocess()` for quality control with a stringent minimum library-size filter (`min_sample_counts = 10000`). We applied multiple transformations (`rlog`, `vst`, `tmm`, and plasmid-feature scaling such as `rtcas1`) using `recoRdseq.transform()`. For each method, the post-normalization group-wise distributions were visualized by computing, for each group a, per-gene medians $m_{g,a} = \mathrm{median}_{i\in a}(T_{g,i})$ from the transformed matrix T (the `normCounts` assay) and plotting the density of $\{m_{g,a}\}_{g=1}^G$ across groups. In parallel, `recoRdseq.transform()` reported distribution-similarity warnings when pairwise group comparisons exceeded either a KS threshold or a Wasserstein-1 (L_1 EMD) threshold (Section S1.2.3).

S1.3.2. Controlled downsampling benchmark under depth asymmetry

To stress-test robustness under controlled, asymmetric sparsity, we constructed a downsampled version of the `Chow` vs `Starch` contrast from the bundled dataset by explicitly resampling reads within each sample to a target library size that differs by group. Let $\mathbf{x}_i = (x_{1,i}, \dots, x_{G,i})^\top$ be the observed count vector for sample i, with $C_i = \sum_g x_{g,i}$. For each repetition, a target depth L_i was drawn uniformly at random within a group-specific range: $L_i \sim \mathrm{Unif}\{5000, \dots, 10000\}$ for `Starch` samples and $L_i \sim \mathrm{Unif}\{25000, \dots, 40000\}$ for `Chow` samples, with $L_i \leq C_i$ enforced. Downsampled counts were generated via multinomial resampling

$$\mathbf{x}_i' \sim \mathrm{Multinomial}(L_i,\ \mathbf{p}_i), \qquad p_{g,i} = \frac{x_{g,i}}{\sum_{h=1}^G x_{h,i}}. \tag{22}$$

This procedure preserves each sample's empirical feature composition while reducing depth to a controlled target, thereby increasing sparsity in the low-depth group. Downsampling was repeated 10 times with different random seeds.

On the downsampled matrix, we ran DESeq2/edgeR/Wilcoxon with identical modelling choices and thresholds (padj < 0.1 and $|\log_2 \mathrm{FC}| \geq 1$). To quantify the impact of downsampling on calls, we defined a shared full-data ground truth set as the three-method intersection from the non-downsampled analysis,

$$\mathcal{G} = \mathcal{C}_{\mathrm{DESeq2}}^{\mathrm{full}} \cap \mathcal{C}_{\mathrm{edgeR}}^{\mathrm{full}} \cap \mathcal{C}_{\mathrm{wilcoxon}}^{\mathrm{full}}, \tag{23}$$

and evaluated each downsampled call set $\mathcal{C}_m^{\mathrm{down}}$ by

$$\mathrm{TP}_m = \left|\mathcal{C}_m^{\mathrm{down}} \cap \mathcal{G}\right|, \quad \mathrm{FP}_m = \left|\mathcal{C}_m^{\mathrm{down}} \setminus \mathcal{G}\right|, \quad \mathrm{FN}_m = \left|\mathcal{G} \setminus \mathcal{C}_m^{\mathrm{down}}\right|, \tag{24}$$

with true-positive rate (TPR) $\mathrm{TP}_m/|\mathcal{G}|$ and false-positive rate (FPR) $\mathrm{FP}_m/(G - |\mathcal{G}|)$ reported when denominators are non-zero. This benchmark is designed to isolate the effects of increased sparsity and distributional mismatch on inference.

S1.3.3. Synthetic simulation benchmark comparing parametric and Wilcoxon strategies

To compare methods under a controlled data-generating process that deliberately violated negative-binomial model assumptions,

we executed a distribution-free simulation stress test using fully synthetic data. Each experimental replicate contained $G = 1000$ genes and $n = 40$ samples split evenly between groups A and B ($n_A = n_B = 20$). Group B possessed an approximately two-fold larger library size than group A. We simulated baseline gene intensities μ_g and sample-level library-size factors ℓ_i as follows:

$$\mu_g = \exp(\eta_g), \qquad \eta_g \sim \mathcal{N}(2.0,\, 0.9^2) \tag{25}$$

$$\ell_i = \begin{cases} \exp(\varepsilon_i), & i \in \mathrm{A} \\ 2\,\exp(\varepsilon_i), & i \in \mathrm{B} \end{cases}, \qquad \varepsilon_i \sim \mathcal{N}(0.1,\, 0.2^2) \tag{26}$$

We injected additional multiplicative sample noise at the gene-by-sample level using $\nu_{g,i} = \exp(\zeta_{g,i})$ with $\zeta_{g,i} \sim \mathcal{N}(0, 0.35^2)$.

We evaluated two scenarios: `signal_plus_outliers` and `global_null`. In `signal_plus_outliers`, we randomly selected a fraction $\pi_{\mathrm{DE}} = 0.12$ of genes as truly differential ($\mathcal{D}$). For each $g \in \mathcal{D}$, we sampled the group-B multiplicative effect once per gene as $e_g = 2^{\Delta_g}$ with $\Delta_g \sim \mathcal{N}(1.5, 0.2^2)$. In `global_null`, we set $\mathcal{D} = \varnothing$ and $e_g = 1$. We generated pre-dropout Poisson counts $X_{g,i}$ from intensity $\lambda_{g,i}$:

$$\lambda_{g,i} = \mu_g\, \ell_i\, \nu_{g,i}\, e_g^{\mathbb{I}[i \in \mathrm{B}]} \tag{27}$$

$$X_{g,i} \sim \mathrm{Poisson}\big(\max(\lambda_{g,i},\, 10^{-6})\big) \tag{28}$$

To induce structured zero inflation, we applied gene- and sample-dependent dropout. We sampled baseline propensities $\pi_g \sim \mathrm{Beta}(1.8, 4.2)$ and set sample-level shifts s_i to 0.08 for group A and −0.05 for group B. We clipped dropout probabilities $d_{g,i}$ to $[0.01, 0.95]$ and generated observed counts $Y_{g,i}$ via a Bernoulli process:

$$d_{g,i} = \mathrm{clip}(\pi_g + s_i;\ 0.01,\ 0.95) \tag{29}$$

$$Y_{g,i} = X_{g,i}(1 - Z_{g,i}), \qquad Z_{g,i} \sim \mathrm{Bernoulli}(d_{g,i}) \tag{30}$$

We forced the first 150 genes to be detected in all samples to improve normalization stability. Finally, we modeled sparse outlier bursts by multiplying null gene counts by a factor of 60 in one random group B sample (8% of non-DE genes).

Across multiple simulations (8 for `signal_plus_outliers`; 6 for `global_null`), we ran `recoRdseq.DE()` (DESeq2 Wald, edgeR QL, and Wilcoxon) on the synthetic `genomeCounts` using thresholds padj < 0.1 and $|\log_2 \mathrm{FC}| \geq 1$. We summarized performance for each method m using the following metrics:

$$\mathrm{TP}_m = |\mathcal{C}_m \cap \mathcal{D}|, \quad \mathrm{FP}_m = |\mathcal{C}_m \setminus \mathcal{D}| \tag{31}$$

$$\mathrm{TPR}_m = \frac{\mathrm{TP}_m}{|\mathcal{D}|}, \quad \mathrm{FPR}_m = \frac{\mathrm{FP}_m}{G - |\mathcal{D}|} \tag{32}$$

The `global_null` scenario provided a direct false-positive assessment. Wilcoxon calibration used shared-label permutations with a reduced budget of 300 per replicate while retaining the strategy from Section S1.2.4.

S1.4. Methods for quantifying local spacer acquisition biases

S1.4.1. Detailed construction of normalized spacer coverage profiles

Genome-aligned spacers from multiple Record-seq experiments were aggregated to construct genome-wide spacer coverage profiles for *E. coli* K-12 MG1655. For each experiment, spacer counts were summed per genomic position to obtain a raw coverage profile,

$$\mathrm{coverage}(x) = \sum_{i=1}^{N_{\mathrm{samples}}} \mathrm{spacers}_i(x), \tag{33}$$

where $\mathrm{spacers}_i(x)$ denotes the number of spacers from sample i mapping to genomic position x.

Raw coverage profiles were globally normalized to a fixed expected library size of 10^7 aligned spacer reads,

$$\mathrm{coverage}_{\mathrm{glob.norm}}(x) = \mathrm{coverage}(x) \cdot \frac{10^7}{\sum_x \mathrm{coverage}(x)}. \tag{34}$$

To reduce expression-driven variation across TUs, globally normalized coverage was locally rescaled by an estimate of TU expression. For a genomic position x mapping to TU TU, normalized coverage was defined as

$$\mathrm{coverage}_{\mathrm{norm}}(x) = \mathrm{coverage}_{\mathrm{glob.norm}}(x) \cdot \frac{1}{\overline{\mathrm{RPKM}}(TU)}, \tag{35}$$

where $\overline{\mathrm{RPKM}}(TU)$ denotes the average RPKM of TU across all samples within the experiment. For positions overlapping multiple TUs, expression estimates were summed, corresponding to fractional feature assignment.

S1.4.2. Similarity metrics for normalized acquisition profiles

Similarity of normalized spacer acquisition profiles between experiments was quantified using complementary metrics capturing positional concordance of localized enrichment and overall profile similarity.

Acquisition hotspots were identified on normalized coverage profiles using the `find_peaks` function from SciPy, with fixed thresholds on peak prominence (0.05) and peak width (10 positions, measured at half-prominence). For a given pair of profiles, peaks in one profile were treated as predictions and peaks in the other as observations. Let TP, FP, and FN denote the numbers of overlapping, non-overlapping predicted, and missing peaks, respectively. Positional agreement was quantified using a peak-level F1 score,

$$\mathrm{F1}_{\mathrm{peak}} = \frac{2\,\mathrm{TP}}{2\,\mathrm{TP} + \mathrm{FP} + \mathrm{FN}}. \tag{36}$$

Overall similarity of acquisition patterns was quantified using the Spearman rank correlation between log-transformed normalized coverage profiles,

$$\rho_{\log} = \mathrm{corr}_{\mathrm{Spearman}}(\log_2(Y + \varepsilon), \log_2(Y' + \varepsilon)), \tag{37}$$

where Y and Y' denote normalized spacer coverage profiles from two experiments and ε is a small pseudocount added for numerical stability.

Baseline.

To establish a baseline for similarity metrics expected by chance, we computed peak-level F1 scores and correlation coefficients between unrelated TUs. Specifically, we randomly sampled pairs of distinct TUs from each dataset and compared their normalized spacer coverage profiles. For each pair, padded positions were

removed independently, profiles were truncated to the minimum shared length, and similarity metrics were computed using the same peak-calling parameters (prominence 0.05, width 10) and overlap criteria as in the main analysis. This procedure was repeated for 100,000 random TU pairs, yielding empirical background distributions for peak-level F1 scores and correlation metrics.

Dataset similarity calculations.
Pairwise similarity across experiments was computed by comparing normalized spacer coverage profiles for matching TUs present in both datasets. For each pair of Record-seq experiments, TUs were matched by genomic coordinates $(start, end)$, and only TUs present in both experiments were retained. Coverage profiles were aligned by genomic position, and similarity metrics were computed across all shared TUs using the same peak-level F1 score and log-Spearman correlation definitions as above. Reported values correspond to averages across all matched TUs for a given experiment pair.

S1.5. Methods for sequence-based modelling of spacer acquisition

S1.5.1. Construction of modelling datasets

To obtain a single normalized coverage profile per TU for modelling, experiment-specific profiles were combined by a weighted average,

$$\text{coverage}_{\text{norm,avg}}(x) = \sum_{e=1}^{E} \frac{R_e}{\sum_{e'=1}^{E} R_{e'}} \text{coverage}_{\text{norm},e}(x), \quad (38)$$

where R_e denotes the total number of aligned spacer reads in experiment e and $\text{coverage}_{\text{norm}}(x)$ is the normalised spacer coverage at genome position x as defined in S1.4.1.

S1.5.2. Formal problem formulation and model inputs

Model inputs consisted of (i) a one-hot encoding of the DNA sequence and (ii) annotation channels encoding transcription-related genomic features, including gene bodies, promoter and terminator locations, and gene orientation. Because TUs vary in length, inputs and targets were grouped into length-matched batches and padded to the maximum TU length within each batch.

Spacer acquisition was modelled as a Poisson process, where each transcribed position i is associated with a constant sampling rate λ_i. Note that because Y_i represents a locally rescaled, relative acquirability rate aggregated across multiple samples—rather than raw, absolute transcript counts that are subject to biological overdispersion from phenomena like transcriptional bursting—the Poisson assumption of equidispersion is statistically appropriate for this continuous target. For these normalized spacer counts Y_i, we assume

$$P(Y_i = k) = \frac{\lambda_i^k e^{-\lambda_i}}{k!}, \qquad i \in \{1, \ldots, n\}, \quad (39)$$

where n denotes the number of transcribed positions within a TU. A neural network f_θ predicts position-specific rates $\hat{\lambda}_i = f_\theta(x_i)$ from sequence and annotation features x_i.

Model parameters were optimized by minimizing the Poisson negative log-likelihood, an approach established by sequence-to-activity models such as Basenji for predicting normalized, base-resolution genomic coverage (Kelley et al. 2018). The loss is defined as:

$$\mathcal{L}(\theta) = \sum_{i=1}^{n} \left(\hat{\lambda}_i - Y_i \log(\hat{\lambda}_i + \varepsilon) \right), \quad (40)$$

where ε is a small constant added for numerical stability. For padded batches, losses were computed with masking to ignore padded positions,

$$\mathcal{L}_{\text{mask}}(\theta) = \sum_{i=1}^{n} M_i \left(\hat{\lambda}_i - Y_i \log(\hat{\lambda}_i + \varepsilon) \right), \quad (41)$$

where $M_i \in \{0, 1\}$ indicates valid (unpadded) positions.

S1.5.3. Genome-wide data splitting and model training procedure

Train/validation/test splits were generated genome-wide at TU resolution while enforcing zero genomic overlap between splits. Each TU was represented by its genomic interval (s, e), with coordinates normalized to ensure $s \leq e$. Overlap-connected components were constructed using a sweep-line procedure under a strict overlap criterion, whereby two intervals were considered connected if $s_{i+1} < e_i$. This guarantees that any TUs sharing at least one genomic position are assigned to the same component, and splits were therefore performed at the component level rather than per TU to prevent sequence leakage.

To ensure genome-wide coverage, components were stratified by their genomic midpoint, defined as

$$m_{\text{comp}} = \frac{\min(s_{\text{comp}}) + \max(e_{\text{comp}})}{2},$$

and binned into $n_{\text{bins}} = 20$ equally spaced bins across the genome. Test and validation sets were assembled by sampling whole components in a stratified manner across bins until the target split fractions were reached, with validation components explicitly excluded from the test set. All remaining components were assigned to the training set, and TU indices within each split were shuffled. The resulting split definition was saved and reused to ensure consistent train/validation/test splits across all datasets.

Model training used the Adam optimizer (learning rate 10^{-3}) with batch size 64. During model selection (`tune_on_val`), models were trained on the training set and monitored on the validation set with early stopping on validation loss. Final evaluation retrained the selected configuration on the combined training and validation data for 60 epochs across five random seeds and results were reported as mean and standard error across the five runs. To improve training stability and computational efficiency, the training and validation sets were restricted to TUs with lengths between 100 and 5,000 nucleotides, excluding approximately 6% of TUs. This length-based filtering was applied only during model selection and training. Validation performance used for early stopping and hyperparameter selection, as well as final model evaluation, was computed on the full validation and test sets, respectively, including all TUs irrespective of length. Model generalizability across the full length distribution was thus assessed on the unfiltered test set.

S2. Supplementary results

S2.1. Additional runtime analyses of the primary analysis pipeline

S2.1.1. PatternBoundExtractor: wavefront-based spacer extraction

To isolate the contribution of the quasi-local alignment strategy, we repeated the runtime benchmarks using a WFA2-based implementation that does not employ length-binned aligners with free end gaps. Under this configuration, runtimes increased substantially with read length and mismatch burden, despite using the same wavefront alignment algorithm. In contrast to the quasi-local formulation used in the main pipeline, alignment costs were no longer restricted to mismatches within the repeat, leading to markedly reduced performance for longer reads and higher error rates (Supplementary Fig. S1).

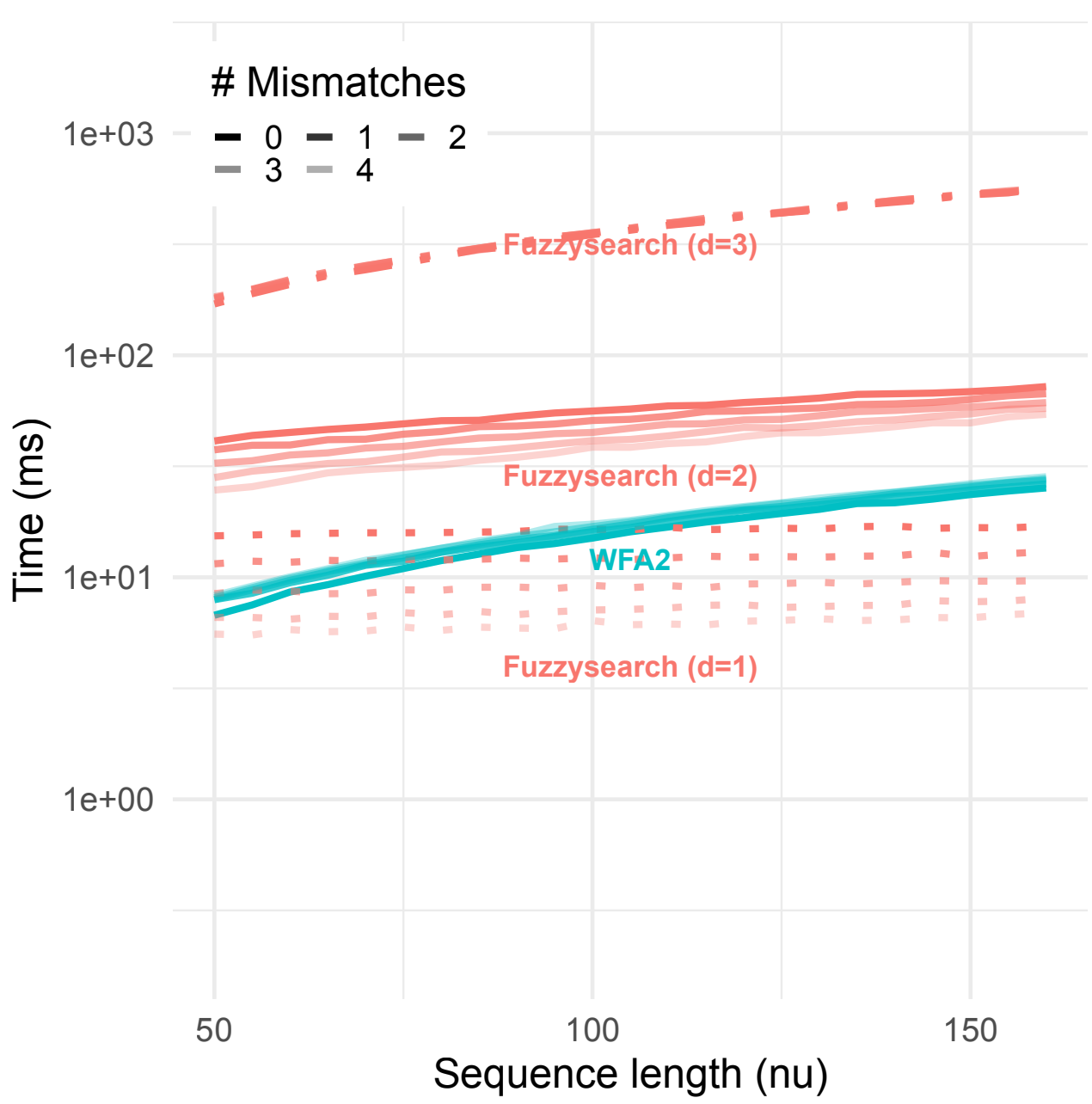

Fig. S1: **Runtime performance of WFA2-based spacer extraction without quasi-local alignment.** Runtime required to perform 1,000 spacer-alignment operations as a function of read length, comparing the original `fuzzysearch`-based implementation across maximum edit-distance thresholds of 1–3 with a WFA2-based spacer extraction approach that does not use length-binned aligners or free end gaps. Benchmarks were performed on synthetic reads containing 0–4 mismatches relative to the pattern. Values represent averages across five independent runs.

S2.2. Secondary analysis benchmarks using `recoRdseq` on real, downsampled, and simulated data

S2.2.1. Distributional diagnostics for normalization and transformation

We reanalyzed the day-7 mouse diet Record-seq dataset from Schmidt et al. (2022) (Chow, Fat, Starch). After preprocessing (library-size filtering and replicate-aware low-depth outlier removal; Supplementary Methods, Section S1.2.2), we compared transformations using `recoRdseq.transform()` distribution-similarity diagnostics (KS and Wasserstein-1/EMD on per-feature group-median profiles; Supplementary Methods, Section S1.2.3).

`rlog` kept all pairwise comparisons below the default warning thresholds (KS $D \leq 0.073$ and EMD ≤ 0.276; Supplementary Fig. S2A). In contrast, `vst` triggered a localized KS warning (Chow vs Fat $D = 0.461$), and `tmm` produced severe mismatches (KS up to 0.513; EMD up to 2.606). We therefore used `rlog` for visualization and QC in this dataset. Applying the same diagnostics to the synthetic simulations used below (Supplementary Methods, Section S1.3.3) yielded the same qualitative ranking (not shown).

S2.2.2. Differential expression on full-depth data

We then performed DE testing on raw `genomeCounts` with `recoRdseq.DE()` for the Chow vs Starch contrast using `DESeq2` (Wald), `edgeR` (quasi-likelihood), and the Wilcoxon workflow (Supplementary Methods, Section S1.2.4). Using padj < 0.1 and $|\log_2 \mathrm{FC}| \geq 1$, all three methods agreed on a shared core of 696 features (Supplementary Fig. S2B). `edgeR` returned the largest call set ($n = 1276$; 271 unique features), `DESeq2` called an intermediate number ($n = 987$; 1 unique), and Wilcoxon called fewer features overall ($n = 799$; 74 unique).

S2.2.3. Downsampling benchmark under depth asymmetry

To induce controlled sparsity mismatch, we downsampled one condition to lower depth (Supplementary Methods, Section S1.3.2; 10 repetitions with different random seeds) and treated the full-depth three-method intersection as a conservative proxy set for evaluation. Wilcoxon was the most conservative method (lowest FPR) with reduced TPR, whereas `DESeq2` and `edgeR` increased TPR at the cost of higher FPR (Supplementary Fig. S2C).

S2.2.4. Simulation benchmark with sparse outlier contamination

We simulated sparse Poisson-lognormal counts with gene- and sample-dependent dropout and sparse outlier bursts to violate negative-binomial assumptions (Supplementary Methods, Section S1.3.3). Across replicates, Wilcoxon achieved the highest TPR with an FPR comparable to `DESeq2`, whereas `edgeR` showed substantially higher FPR (Fig. 3). In a global-null setting, `edgeR` produced the largest false-positive burden.

S2.3. Additional results for sequence-based acquisition modelling

S2.3.1. Architecture and sequence-feature sensitivity analyses

To further interpret how models recover local acquisition structure, we examined architecture ablations and sequence-feature sensitivity. Convolution-only architectures performed substantially worse than recurrent models, and adding dot-product attention to the recurrent baseline did not improve performance (not shown). Convolutional filters did not yield readily interpretable motifs, and performance did not improve with larger receptive fields, arguing against a dominant role for motif-driven recognition. Small convolutional filters (kernel size 2) in the feature extraction layer were optimal, indicating reliance on short-range patterns integrated over longer contexts by recurrence. Regions with high observed and predicted acquisition were AT-rich, and local GC fraction was inversely correlated with

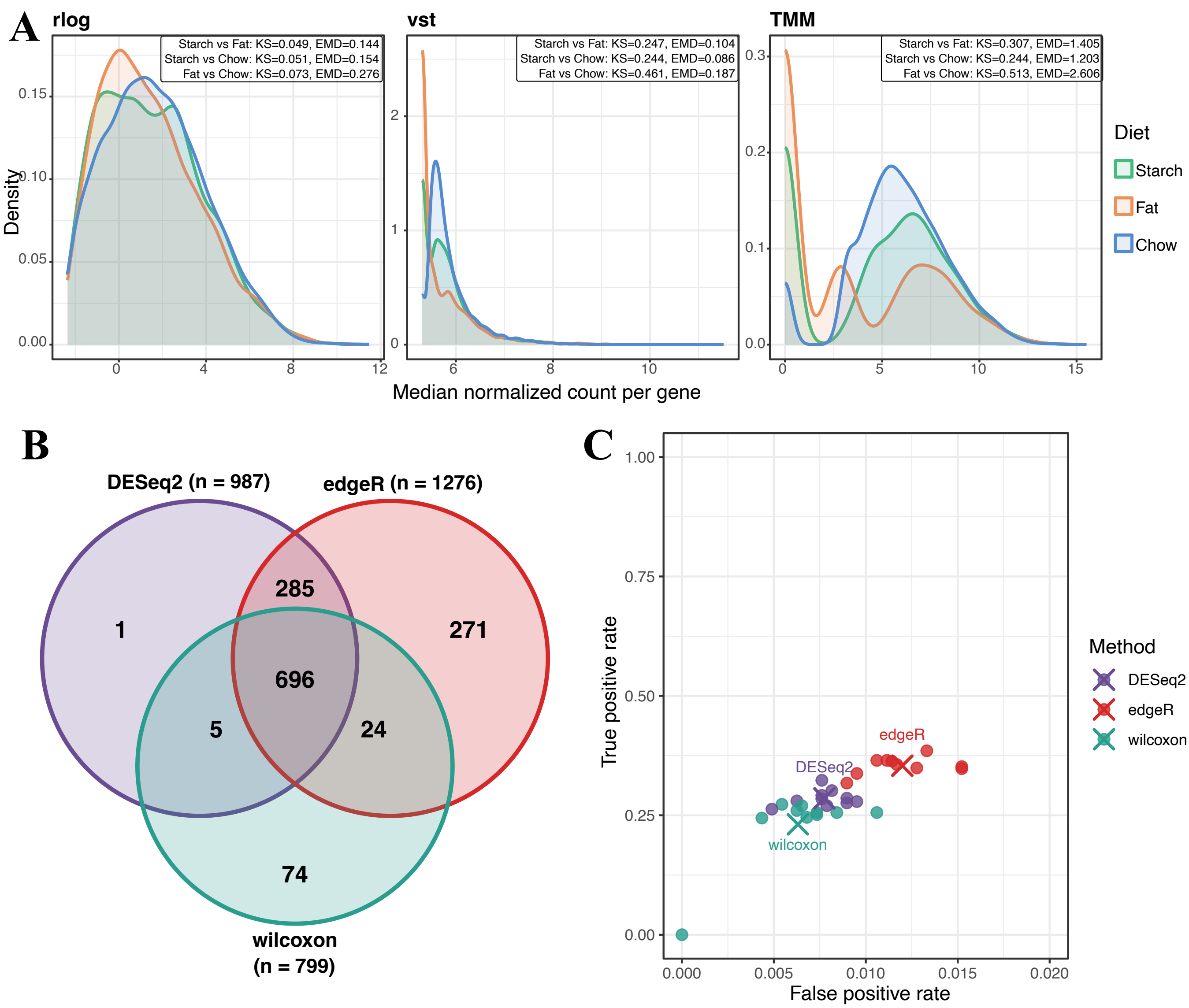

Fig. S2: **Secondary analysis benchmarks using `recoRdseq` on real and downsampled data.** **(A)** Post-transformation distributional comparability across diet groups (Chow, Fat, Starch) for `rlog`, `vst`, and `tmm`. Densities show per-feature group medians computed from `normCounts`; insets report pairwise Kolmogorov–Smirnov (KS) statistics and Wasserstein-1 (EMD) distances between group-median vectors. **(B)** Overlap of differential-abundance calls for the Chow vs Starch contrast across `DESeq2` (Wald), `edgeR` (quasi-likelihood), and the `recoRdseq` Wilcoxon workflow using padj < 0.1 and $|\log_2 \mathrm{FC}| \geq 1$. **(C)** Downsampling benchmark inducing group-wise depth asymmetry (10 repetitions): ROC summary (TPR vs FPR) for each method evaluated against a conservative proxy set defined as the full-depth three-method intersection. Simulation ROC outcomes (TPR vs FPR) are shown in Fig. 3.

acquisition propensity (Spearman $\rho \approx -0.48$), suggesting that base composition captures sequence properties such as accessibility or flexibility relevant for spacer acquisition.

To complement the example in Fig. 4B, we visualized observed and predicted normalized spacer acquisition profiles for 32 transcription units (TUs) randomly sampled from the held-out test set in Supplementary Fig. S3 and S4. The former shows predictions from the model trained on both sequence and annotation streams, while the latter shows the model trained on sequence data alone. For both figures, we visualized the model instance with the median performance, calculated by z-scoring and averaging the Spearman correlation of log-transformed profiles and the peak-level F1 score across the five models trained with different random seeds.

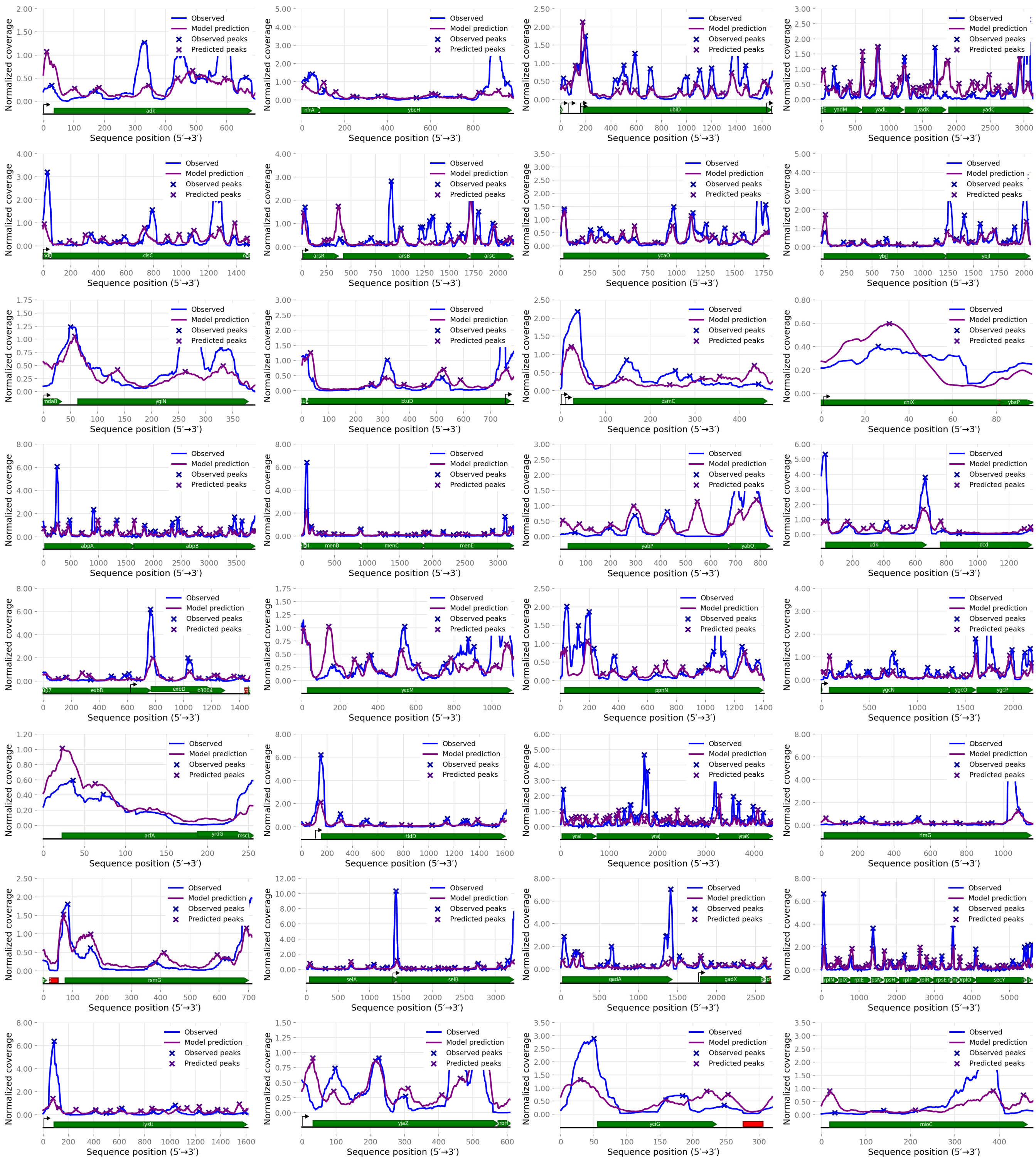

Fig. S3: **Observed and predicted spacer acquisition profiles across representative TUs.** Observed normalized spacer acquisition profiles (blue) and corresponding predictions from the full sequence-and-annotation model (purple) are shown for 32 representative TUs held out during training. Profiles were averaged across experiments with weights proportional to the number of aligned spacer reads. Local acquisition hotspots identified by the peak-calling procedure are indicated by crosses. Gene bodies are shown as green boxes and promoter locations are indicated by black arrows.

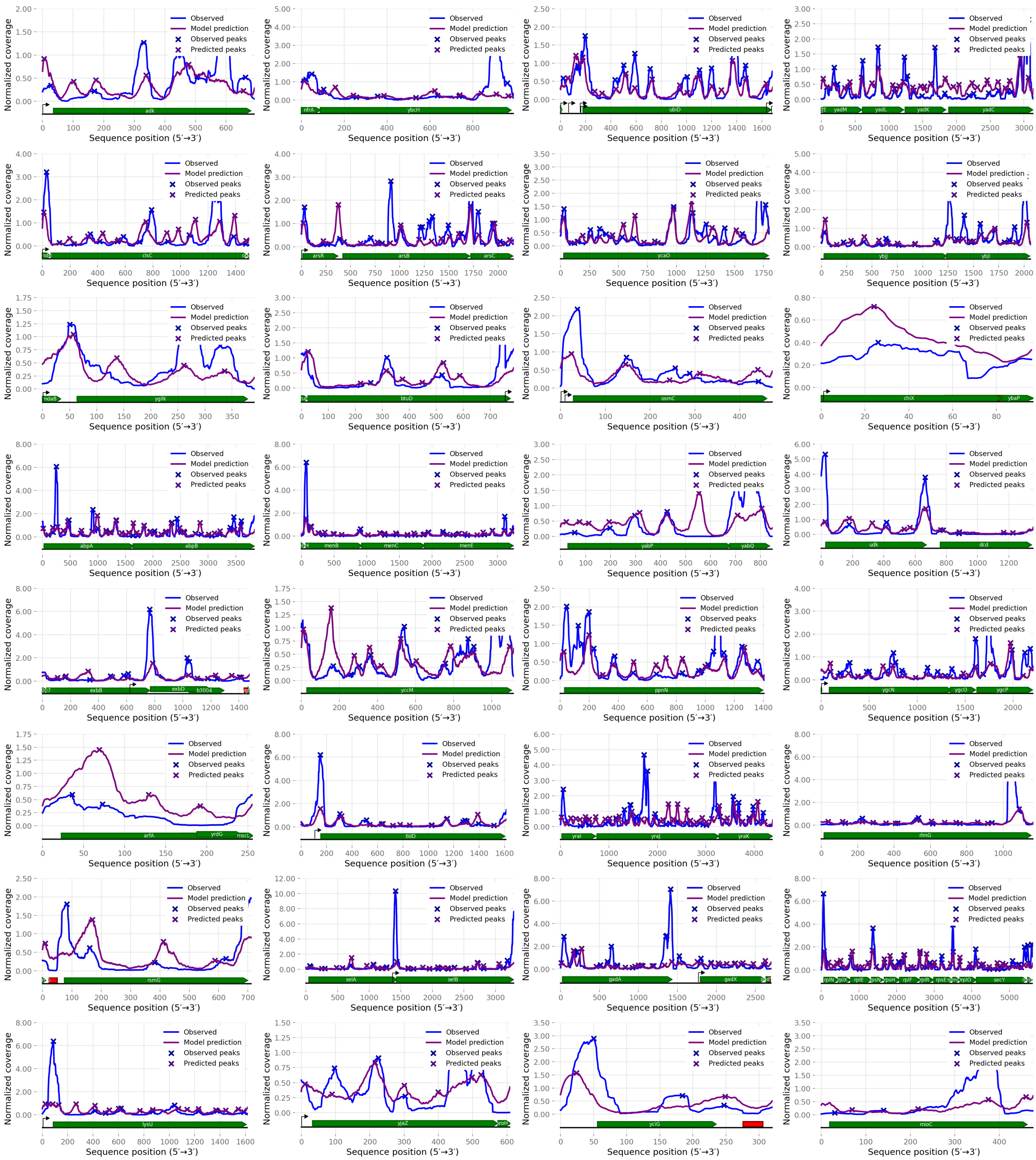

Fig. S4: **Observed and predicted spacer acquisition profiles across representative TUs using a sequence-only model.** Observed normalized spacer acquisition profiles (blue) and predictions from a model trained using DNA sequence information alone (purple) are shown for the same set of 32 TUs as in Supplementary Fig. S3. Profiles were averaged across experiments with weights proportional to the number of aligned spacer reads. Local acquisition hotspots identified by the peak-calling procedure are indicated by crosses. Gene bodies are shown as green boxes and promoter locations are indicated by black arrows.

References

Amitai G, Sorek R. CRISPR–Cas adaptation: insights into the mechanism of action. Nat Rev Microbiol 2016;14:67–76.

Cleal K. pyWFA: Python wrapper for wavefront alignment using WFA2-lib [software]. Version 0.5.1. GitHub, 2024. `https://github.com/kcleal/pywfa` (29 June 2026, date last accessed).

Einat T. fuzzysearch: Approximate string matching for Python [software]. Version 0.8.1. PyPI/GitHub, 2025. `https://github.com/taleinat/fuzzysearch` (29 June 2026, date last accessed).

Karp PD, Paley S, Caspi R et al. The EcoCyc database (2025). EcoSal Plus 2025;13:eesp-0019-2024. doi:10.1128/ecosalplus.esp-0019-2024.

Kelley DR, Reshef YA, Bileschi M et al. Sequential regulatory activity prediction across chromosomes with convolutional neural networks. Genome Res 2018;28:739–50.

Liao Y, Smyth GK, Shi W. featureCounts: an efficient general purpose program for assigning sequence reads to genomic features. Bioinformatics 2014;30:923–30.

Love MI, Huber W, Anders S. Moderated estimation of fold change and dispersion for RNA-seq data with DESeq2. Genome Biol 2014;15:550.

Mao X, Ma Q, Liu B et al. Revisiting operons: an analysis of the landscape of transcriptional units in *E. coli*. BMC Bioinformatics 2015;16:356.

Marco-Sola S, Moure JC, Moreto M, Espinosa A. Fast gap-affine pairwise alignment using the wavefront algorithm. Bioinformatics 2021;37:456–63.

Robinson MD, McCarthy DJ, Smyth GK. edgeR: a Bioconductor package for differential expression analysis of digital gene expression data. Bioinformatics 2010;26:139–40.

Schmidt F, Cherepkova MY, Platt RJ. Transcriptional recording by CRISPR spacer acquisition from RNA. Nature 2018;562:380–5.

Schmidt F, Zimmermann J, Tanna T et al. Noninvasive assessment of gut function using transcriptional recording sentinel cells. Science 2022;376:eabm6038.

Tanna T, Schmidt F, Cherepkova MY et al. Recording transcriptional histories using Record-seq. Nat Protoc 2020;15:513–39.

Xu J, Bjursell MK, Himrod J et al. A genomic view of the human-*Bacteroides thetaiotaomicron* symbiosis. Science 2003;299:2074–6.
